# Handgrip Strength Reference Values and Compositional Associations with Physical Activity in Early Childhood: A Large Sample Study of Swedish Preschoolers

**DOI:** 10.1186/s40798-026-00992-4

**Published:** 2026-02-27

**Authors:** Ana Ramírez-Osuna, Pablo Campos-Garzón, Francisco Javier Huertas-Delgado, Viktor H. Ahlqvist, Charlotte Wilén, Pontus Henriksson, Tommy R. Lundberg, Martin Neovius, Micael Dahlen, Daniel Berglind

**Affiliations:** 1 Department of Physical Education and Sports, Faculty of Sport Sciences, Sport and Health University Research Institute (iMUDS), University of Granada, Granada, Spain; 2 Department of Global Public Health, Karolinska Institutet, Stockholm, Sweden; 3 Faculty of Health Sciences, University of Lethbridge, Lethbridge, AB Canada; 4 “La Inmaculada” Teacher Training Centre, University of Granada, Granada, 18013 Spain; 5 Department of Biomedicine, Aarhus University, Aarhus, Denmark; 6 Institute of Environmental Medicine, Karolinska Insitutet, Stockholm, Sweden; 7 Department of Medical and Health Sciences, Linköping University, Linköping, 581 83 Sweden; 8 Division of Clinical Physiology, Department of Laboratory Medicine, Karolinska Institute, Stockholm, Sweden; 9 Unit of Clinical Physiology, Karolinska University Hospital, Stockholm, Sweden; 10 Centre for Epidemiology and Community Medicine, Region Stockholm, Stockholm, Sweden

**Keywords:** Muscular strength, Reference values, Preschoolers, Physical fitness

## Abstract

**Background:**

Muscular strength is a marker of current health and a predictor of long-term health outcomes in young populations, supporting the inclusion of muscle-strengthening activities into current guidelines and recommendations. Over the last decade, muscular strength has been included in several fitness-test batteries in children and adolescents. However, little is known about its relevance and the feasibility of assessing it in preschool children aged 3–5 years. Therefore, in this cross-sectional study, we aimed to generate reference values for handgrip strength in Swedish preschool children and to examine the associations of device-measured movement behaviours (sedentary time [ST], light physical activity [LPA], moderate-to-vigorous physical activity [MVPA], and sleep duration) with handgrip strength using compositional data analysis.

**Results:**

A total of 3,218 preschool children (48.53% female) aged 3.0–5.5 years from Sweden were included. Handgrip strength was measured using a validated analog dynamometer following standardized procedures. Movement behaviours were assessed in a subsample of 2,328 children who had both handgrip data and valid accelerometer recordings. Compositional data analysis was used to examine associations between handgrip strength and the 24-hour time-use composition, adjusting for age, sex, body mass index, parental education, and wear time. Age- and sex-specific percentiles for handgrip strength were developed. Boys showed higher handgrip values than girls at all ages (e.g., median increased from 4.08 to 7.42 kg in boys and from 3.45 to 6.87 kg in girls between ages 3 and 5 years). When the proportion of time spent in MVPA increased relative to the other behaviours, handgrip strength rose by + 1.22 kg; the opposite was observed for ST, which related to − 0.84 kg lower handgrip strength. No significant associations were observed for LPA or sleep duration (LPA: β =-0.48 kg, 95% CI: -1.23, 0.27; sleep: β = 0.10 kg, 95% CI: -0.37, 0.57).

**Conclusion:**

This study provides the first normative reference values for handgrip strength from Northern Europe. These values offer a useful reference for screening and contextual interpretation of muscular strength in preschool children.

**Supplementary Information:**

The online version contains supplementary material available at 10.1186/s40798-026-00992-4.

## Introduction

Increasing evidence suggests that muscular strength is a marker of current health and a predictor of long-term health outcomes in young populations, [1–3] supporting the inclusion of muscle-strengthening activities into current guidelines and recommendations [4]. Over the last decade, muscular strength has been included in several fitness-test batteries in children and adolescents, and numerous studies have highlighted it as an important marker of health in these age groups [5, 6]. However, little is known about its relevance and the feasibility of assessing it in preschool children aged 3–5 years [6]. A major challenge is the current lack of normative reference values for young children, rendering definitions of poor muscular fitness highly study-specific and limiting comparability across populations.

Most reference values for muscular strength have been established in school-aged children and adolescents, including studies from Hungary [7] and the United States, [8] with very limited data available for preschool-aged children. Recently, Cadenas-Sánchez et al. [9] provided reference values for handgrip strength in Spanish preschool children. However, there is still a lack of data from other regions, particularly from Northern Europe, [10] where such data are essential to enable pooled analyses and the development of European or global reference values for handgrip strength in preschoolers [11].

Establishing normative reference values for handgrip strength in preschool-aged children, alongside investigating its associations with physical activity (PA) patterns, is relevant for the early identification of potential developmental concerns and the design of targeted strategies to support physical health. However, the current lack of data on young children prevents such efforts. To address this gap, we aimed to generate normative reference values for handgrip strength in a large sample of preschool children aged 3–5 years from the Stockholm region. In addition, in the subsample with valid accelerometry, we examined how the 24-hour composition of movement behaviours (sedentary time, light physical activity, moderate-to-vigorous physical activity, and sleep) relates to handgrip strength using compositional data analysis, which accounts for the co-dependence of time-use data [12, 13].

## Methods

### Study Population and Data Source

This cross-sectional study was conducted as part of the Increasing Children’s physical Activity by Policy (CAP) study with reference number: NCT04569578 was reported in accordance with the STROBE guidelines. The aim of the CAP study was to evaluate the feasibility and effectiveness of policy measures to increase PA in preschool children. To ensure adequate representation of the Stockholm region, including areas with different socio-economic status, a stratified clustered sampling design was used to sample preschool children from all 11 districts of Stockholm (85% Swedish preschool children aged 3–5 years old). Briefly, all 3–5-year-old preschool children enlisted in the participating preschools were invited to participate, and participation was confirmed through informed consent from parents. Further details about the recruitment process and methodological procedures of the CAP study can be found elsewhere [14]. Ethical approval was obtained from the Swedish Ethical Review Authority (Dnr. 2020–03002).

### Participants

**Fig. 1:**
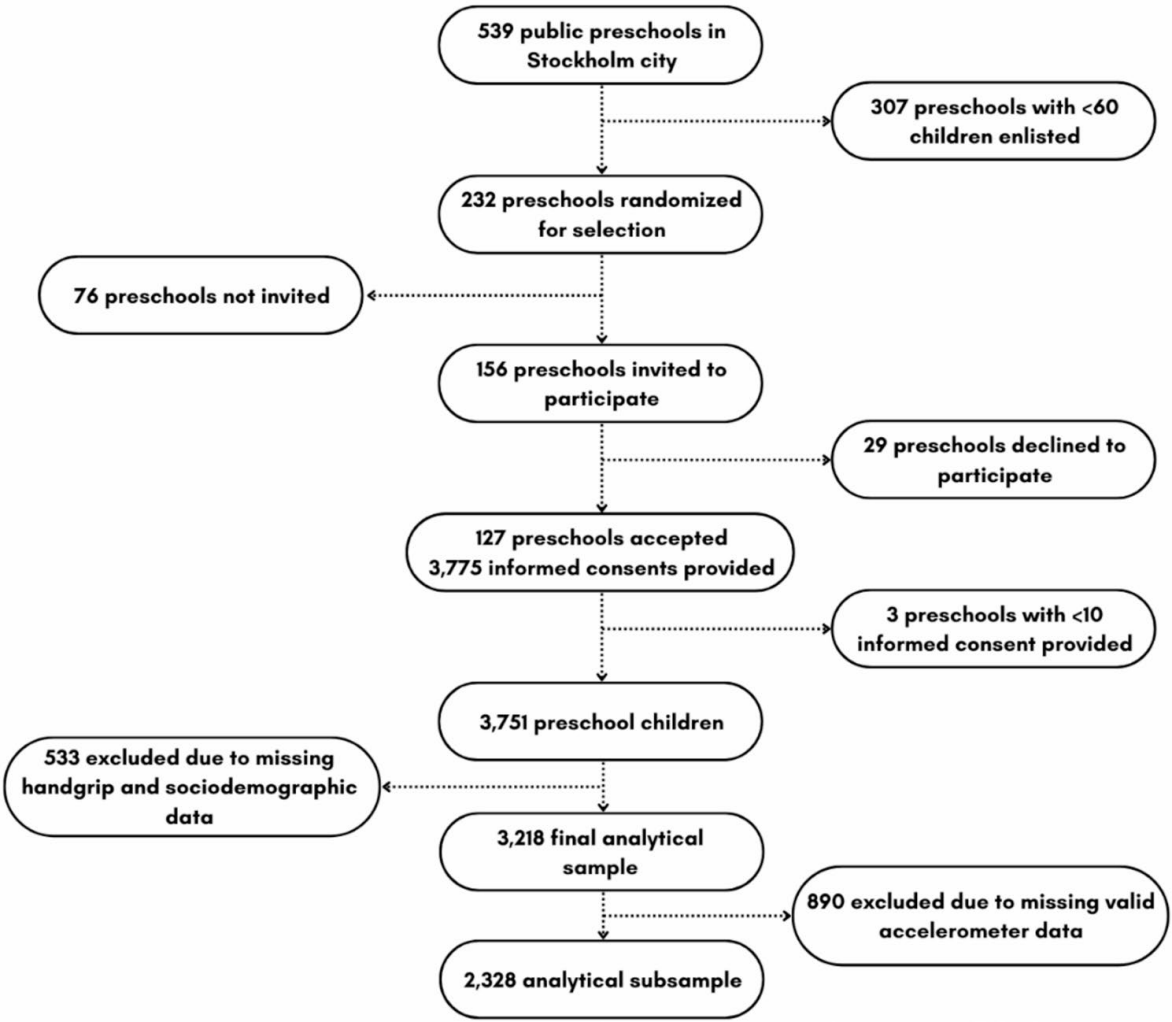
Flowchart of participants

In the current study, data from the CAP study was used in accordance with what is presented in Fig. 1. In Stockholm, 156 public preschools were invited to participate and in total data from 124 participating preschools were collected between September 2020 and February 2021. Signed informed consent was obtained from the parents or legal guardians of 3,401 Swedish preschool children aged 3–5 years. For the establishment of reference values, children were included if they met the following inclusion criteria: (1) valid handgrip strength measurements and (2) complete data on age and sex. In a secondary analysis, the association between handgrip strength and PA was explored in the subsample of children who additionally had valid accelerometer data, defined as having at least one day with ≥ 8 waking hours, and age, sex, body mass index (BMI) and parental education reported (both mother and father). Based on these criteria, a final analytical sample of 3,218 observations was retained for the main analysis. In a secondary analysis, the association between handgrip strength and PA was explored in the subsample of 2,328 preschool children who additionally had valid accelerometer data.

### Measures

#### Demographic Information

Sociodemographic data were collected through a questionnaire completed by the parents, which included information on parental education level. The age and sex of both preschool children and parents were derived from the “Swedish personal identity number” provided at the time of consent. Parental education level was categorized into elementary school, upper secondary school, or university education.

Musculoskeletal fitness (primary outcome).

Musculoskeletal fitness was assessed using handgrip strength, measured with an analog dynamometer (TKK 5825, Grip-A, Takei, Tokyo, Japan) validated for use in preschool-aged children [15]. The measurement protocol followed the PREFIT battery recommendations for preschool children, ensuring standardization and comparability with other studies [6, 16]. Handgrip strength was recorded to the nearest 0.1 kg for both the dominant and non-dominant hand. Preschool children performed two trials per hand, and the highest value was recorded for analysis. For normative reference values, we used the mean of the maximal values obtained from the right and left hand (bilateral mean). For side-specific analyses, the maximal value of each hand was used separately. Trained field staff conducted all measurements following standardized protocols to ensure accuracy and reliability. To ensure consistency, preschool children were instructed to stand upright with their arms extended downward while squeezing the dynamometer as forcefully as possible. Rest intervals were provided between trials to avoid fatigue.

### Accelerometry

Sedentary time (ST), sleep duration, PA levels and steps in preschool children were monitored using triaxial GT3X+ accelerometers. Preschool children were instructed to wear the accelerometer on their non-dominant wrist continuously (24 h per day) for seven consecutive days, except during water-based activities. The non-dominant hand was reported by the parents (information was collected with the informed consent). Accelerometer data were set up and analyzed according to validated age-specific criteria and recommendations [17]. The accelerometer sampling frequency was set at 100 Hz, and data were analyzed at 5-s epochs, which corresponds to the sporadic movement pattern of preschool children. The raw accelerometer data were analyzed using the GGIR package [18]. The classification of ST and PA intensities followed the cut-points developed by Hildebrand et al. for preschool children using the wrist-worn Actigraph GT3X+ devices: ST < 35 mg, LPA between 35 and 199 mg and MVPA ≥ 200 mg [19, 20]. Steps were determined using the Verisense step algorithm [21].

### Anthropometry

Weight and height of the participating preschool children were measured by trained staff using validated scales and stadiometers. Age- and sex-specific international body BMI classification was used to categorize preschool children into “normal”, “overweight” and “obesity” categories based on established age-specific normative reference values [16].

### Statistical Analysis

Participants were stratified by age and sex. Descriptive statistics are reported as means ± Standard deviation (SD) for continuous variables and as frequencies and percentages for categorical variables. A Box-Cox transformation was applied to stabilize the variance and approximate a normal distribution. LMS parameters (Lambda for skewness, Mu for median, and Sigma for variability) were estimated using GAMLSS with penalized maximum likelihood to generate age-specific smoothed percentile curves. In addition, as a sensitivity analysis, exploratory allometric models were fitted to examine the scaling of handgrip strength with body size. Log–log mixed-effects models were used to estimate scaling exponents for height and body mass in the full sample and separately by sex. Based on these estimates, size-normalized handgrip strength values were derived using a generalized allometric ratio, and age- and sex-specific percentiles of normalized handgrip strength were calculated. These analyses were conducted to assess the potential influence of body size on the interpretation of handgrip strength reference values and are presented in the Supplementary Material (Tables S5–S7). To analyze associations between handgrip strength and PA levels, compositional data analysis (CoDA) was conducted using isometric log-ratio (ilr) transformations of time-use components, including ST, LPA, MVPA and sleep duration. The CoDA was adjusted for age, sex, BMI, parental education level, and accelerometer wear time. In addition, isotemporal substitution models were implemented to estimate the theoretical change in handgrip strength associated with reallocating fixed durations of time between movement behaviours (ST, LPA, MVPA, and sleep) while keeping total wear time constant. To aid interpretation, a ternary plot of the three waking behaviours (ST, LPA, MVPA) coloured by handgrip tertiles was produced. The significance of fixed effects was assessed with Wald tests, and all statistical analyses were performed in RStudio (version 2024.12.0). Sensitivity analyses included comparisons between descriptive characteristics of included and excluded participants (see Table S1 in Supplementary Material).

## Results

Descriptive data of the participants are shown in Table 1, stratified by sex and age. Among the preschool participants, 45.2% of boys and 45.7% of girls were classified as having normal weight, while 35.4% of boys and 31.6% of girls were classified as underweight according to international BMI normative reference values [16]. Parental education levels were predominantly university level, both for preschool boys (92.6% for fathers and 89.4% for mothers) and preschool girls (93.0% for fathers and 90.2% for mothers). Most preschool children were right-handed, with 90.3% of boys and 91.8% of girls having a dominant right hand. All values are presented below as means ± standard deviations, unless otherwise specified. Handgrip strength increased with age; in boys it averaged 5.2 ± 2.3/4.8 ± 2.2 kg (right/left) at age 3 years and 9.1 ± 2.6/8.8 ± 2.5 kg at age 5 years, and in girls 4.3 ± 2.2/4.0 ± 2.1 kg at age 3 years and 8.2 ± 2.5/7.8 ± 2.4 kg at age 5 years. The preschool children were sedentary for 424.9 ± 78.0 min/day (boys) and 421.7 ± 77.8 min/day (girls). In terms of PA levels, both boys and girls exceeded the recommended 180 min of PA per day on average, with values of LPA ranging from 288 ± 54.2 to 300 ± 47.3 min/day for boys, and from 278 ± 57.7 to 303 ± 48.1 min/day for girls. Regarding MVPA, the mean value in preschool boys aged 5 years reached 65.8 ± 21.5 min/day, exceeding the recommended 60 min of daily PA, while preschool girls were on average below the recommended level at 56.0 ± 17.4 min/day. Step counts indicated that both boys and girls on average exceeded 10,000 steps per day, with boys taking 13,554 ± 2,798 steps/day and girls 12,997 ± 2,702 steps/day on average at age 5.

**Table 1:** Descriptive characteristics of participants

		Boys				Girls		
	All (*n* = 1656)	3 years (*n* = 465)	4 years (*n* = 613)	5 years (*n* = 578)	All (*n* = 1562)	3 years (*n* = 458)	4 years (*n* = 572)	5 years (*n* = 532)
Weight (kg)	19.2 ± 3.0	17.1 ± 2.0	19.0 ± 2.6	21.1 ± 3.0	18.6 ± 3.2	16.5 ± 2.0	18.5 ± 2.6	20.8 ± 3.0
Height (cm)	108.1 ± 7.0	101.1 ± 4.6	107.8 ± 4.6	114.2 ± 4.8	106.6 ± 7.3	99.4 ± 4.5	106.6 ± 4.5	113.3 ± 4.9
Waist (cm)	54.6 ± 4.6	53.8 ± 4.4	54.6 ± 4.6	55.7 ± 4.8	54.3 ± 4.9	53.5 ± 4.5	54.2 ± 4.8	55.6 ± 5.0
BMI (kg/m^2^)	16.3 ± 1.4	16.7 ± 1.2	16.3 ± 1.4	16.1 ± 1.5	16.3 ± 1.5	16.6 ± 1.3	16.2 ± 1.47	16.1 ± 1.6
Normal (n/%)	749(45.2)	227(48.8)	303(49.4)	219(37.9)	714(45.7)	202(44.1)	280(49.0)	232(43.6)
Overweight (n/%)	208(12.6)	103(22.2)	56(9.1)	49(8.5)	213(13.6)	97(21.2)	63(11.0)	53(10.0)
Obese (n/%)	113(6.8)	53(11.4)	38(6.2)	22(3.8)	142(9.1)	78(17.0)	40(7.0)	24(4.5)
Underweight (n/%)	586(35.4)	82(17.6)	216(35.2)	288(49.8)	493(31.6)	81(17.7)	189(33.0)	223(41.9)
Paternal education(n%)								
Elementary	28(2.9)	8(2.8)	6(1.7)	14(4.2)	23(2.5)	3(1.1)	8(2.3)	12(3.9)
Secondary	44(4.5)	12(4.2)	19(5.4)	13(3.9)	42(4.5)	11(3.9)	17(4.9)	14(4.6)
University	903(92.6)	268(93.1)	328(92.9)	307(91.9)	866(93.0)	266(95.0)	321(92.8)	279(91.5)
Maternal education(n%)								
Elementary	45(6.8)	13(6.1)	16(6.8)	16(7.5)	35(5.7)	10(5.2)	14(6.4)	11(5.4)
Secondary	25(3.8)	9(4.2)	4(1.7)	12(5.6)	25(4.1)	7(3.7)	7(3.2)	11(5.4)
University	591(89.4)	190(89.6)	215(91.5)	186(86.9)	552(90.2)	174(91.1)	197(90.4)	181(89.2)
Dominant hand (n%)								
Hand right	1317(90.3)	370(90.0)	481(89.7)	466(90.3)	1279(91.8)	391(93.8)	454(89.9)	434(91.9)
Hand left	142(9.7)	37(9.1)	55(10.3)	50(9.7)	115(8.2)	26(6.2)	51(10.1)	38(8.1)
Handgrip strength (kg)								
Hand right	7.4 ± 2.9	5.2 ± 2.3	7.0 ± 2.5	9.1 ± 2.6	6.5 ± 2.8	4.3 ± 2.2	6.2 ± 2.2	8.2 ± 2.5
Hand Left	7.1 ± 2.9	4.8 ± 2.2	6.7 ± 2.4	8.8 ± 2.5	6.2 ± 2.7	4.0 ± 2.1	5.9 ± 2.1	7.8 ± 2.4
Sedentary time, PA levels and steps								
Wear time (min/day)	769.4 ± 78.2	758 ± 90.2	755 ± 83.8	778 ± 79.0	758.6 ± 77.6	733 ± 94.0	749 ± 81.6	768 ± 69.5
ST (min/day)	424.9 ± 78.0	433 ± 86.7	413 ± 83.4	424 ± 82.1	421.7 ± 77.8	428 ± 95.2	407 ± 75.2	419 ± 73.4
LPA (min/day)	299.7 ± 48.0	288 ± 54.2	295 ± 52.2	300 ± 47.3	297.5 ± 51.0	278 ± 57.7	298 ± 49.9	303 ± 48.1
MVPA (min/day)	57.7 ± 19.7	49.1 ± 18.2	59.1 ± 21.9	65.8 ± 21.5	49.7 ± 16.7	42.1 ± 15.6	52.8 ± 16.9	56.0 ± 17.4
Steps (number/day)	12,812 ± 2736	12,119 ± 2741	13,037 ± 2862	13,554 ± 2798	12,149 ± 2623	11,481 ± 2740	12,641 ± 2870	12,997 ± 2702

Data are represented as mean ± SD for continuous variables and frequency (percentage) for categorical variables. BMI= body mass index; kg= kilograms; cm= centimeters; ST= sedentary time; LPA= light physical activity; MVPA= moderate to vigorous physical activity.

**Fig. 2:**
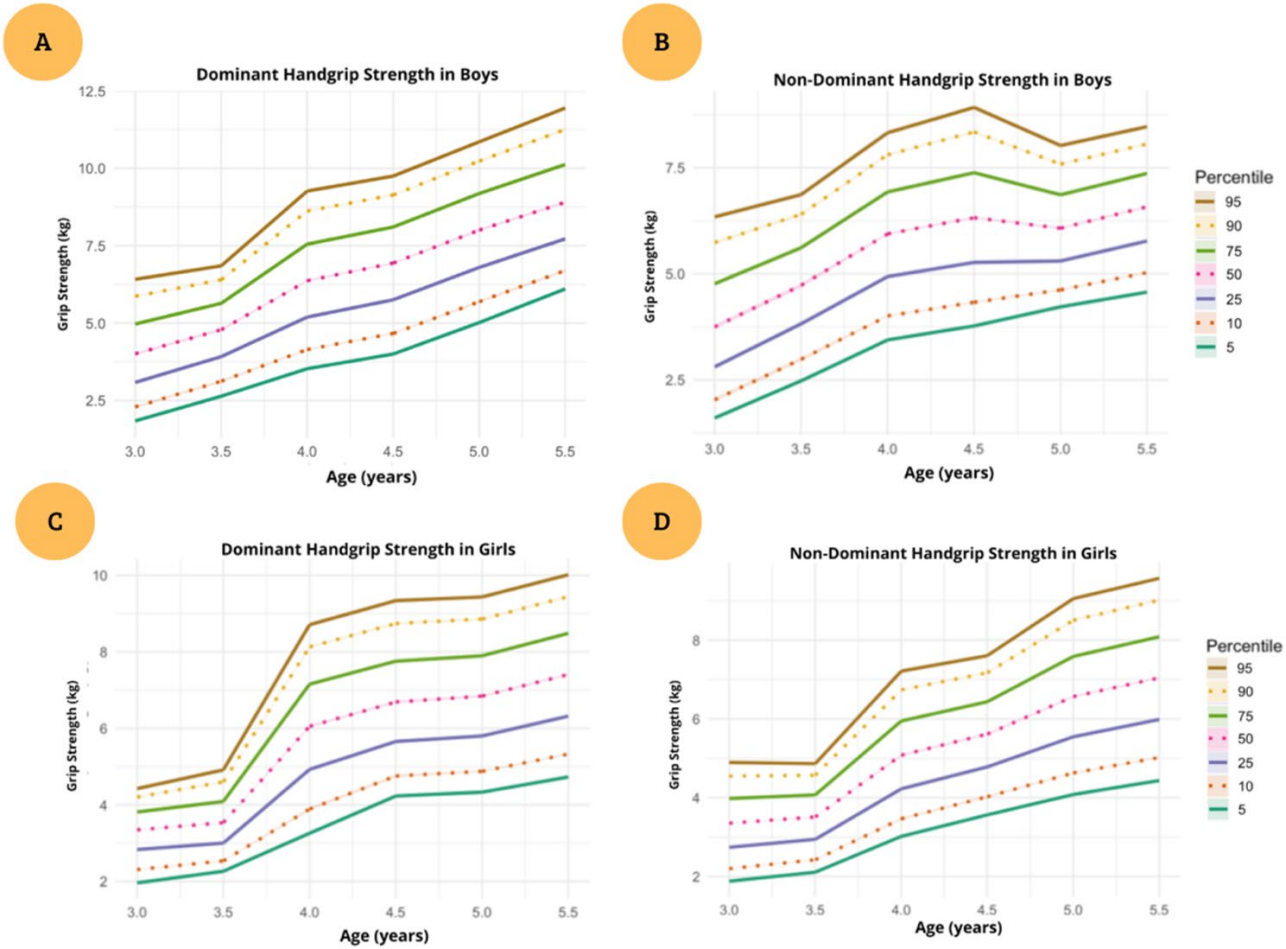
Age- and sex-specific smoothed percentile curves (P5–P95) for handgrip strength in preschool children aged 3.0–5.5 years. Panels show dominant and non-dominant handgrip strength in boys (A–B) and girls (C–D)

The sex-specific reference percentiles derived from the bilateral mean of both hands are summarized in Tables 2 and 3. Percentile curves (P5, P10, P25, P50, P75, P90 and P95) characterize the full distribution of handgrip strength for each 0.5-year age band; percentile values with 95% confidence intervals are provided in Supplementary Tables S2–S4. The corresponding smoothed percentile curves are illustrated in Fig. 2. In boys, the 5th percentile increased by 2.6 times, from 1.81 kg at age 3.0 years to 5.33 kg at age 5.5 years. The median (50th percentile) rose by 1.7 times, from 4.08 kg to 7.42 kg, and the upper tail (95th percentile) increased by 1.3 times, from 6.75 kg to 9.54 kg over the same age span. In girls, the 5th percentile increased by 2.1 times, rising from 2.04 kg at age 3.0 years to 4.58 kg at age 5.5 years. Correspondingly, the median increased by 1.9 times (from 3.45 kg to 6.87 kg) and the 95th percentile by 2.0 times (from 4.55 kg to 9.09 kg).

**Table 2:** Reference standards of hand grip strength (kg) in boy’s preschool children

	P5	P10	P25	P50	P75	P90	P95
3.0	1.81	2.27	3.09	4.08	5.13	6.13	6.75
3.5	2.75	3.27	4.13	5.06	5.96	6.76	7.24
4.0	3.68	4.26	5.22	6.29	7.36	8.31	8.88
4.5	4.09	4.68	5.67	6.78	7.89	8.88	9.48
5.0	4.63	5.11	5.91	6.78	7.65	8.44	8.80
5.5	5.33	5.79	6.56	7.42	8.29	9.07	9.54

Data are presented for 0.5-year age groups; values correspond to the mean hand-grip strength of both hands (kg). P10: 10th percentile; other percentiles are abbreviated accordingly.

**Table 3:** Reference standards of hand grip strength (kg) in girl’s preschool children

	P5	P10	P25	P50	P75	P90	P95
3.0	2.04	2.39	2.92	3.45	3.93	4.33	4.55
3.5	2.31	2.58	3.05	3.58	4.13	4.63	4.94
4.0	3.40	3.89	4.72	5.64	6.58	7.42	7.93
4.5	4.16	4.62	5.40	6.27	7.14	7.92	8.39
5.0	4.25	4.73	5.52	6.40	7.28	8.08	8.55
5.5	4.58	5.09	5.94	6.87	7.79	8.61	9.09

Data are presented for 0.5-year age groups; values correspond to the mean hand-grip strength of both hands (kg). P10: 10th percentile; other percentiles are abbreviated accordingly.

Exploratory allometric analyses showed that handgrip strength scaled more strongly with height than with body mass, with estimated height exponents of 2.49 in boys and 3.33 in girls, and more modest mass exponents (0.52 and 0.36, respectively; Table S5). Although sex-specific differences in scaling exponents were observed, the overall pattern supported the use of common theoretical exponents for size normalization. Based on this approach, age- and sex-specific percentiles of size-normalized handgrip strength were derived (Tables S6 and S7). In boys, the median (50th percentile) increased from 1.61 at age 3.0 years to 2.51 at age 5.5 years, with parallel increases across the lower and upper percentiles. In girls, the median rose from 1.41 at age 3.0 years to 2.25 at age 5.5 years. Across both sexes, size-normalized percentiles showed a consistent age-related increase, indicating that adjustment for body size preserved the expected developmental pattern of increasing muscular strength across the preschool years.

Associations between time-use composition and handgrip strength are presented in Table 4. A higher relative proportion of MVPA was positively associated with strength (β = 1.22 kg; 95% CI 0.81, 1.62), whereas a greater proportion of ST was inversely associated (β = − 0.84 kg; 95% CI − 1.31, − 0.36). LPA (β = − 0.48 kg; 95% CI − 1.23, 0.27) and sleep duration (β = 0.10 kg; 95% CI − 0.37, 0.57) showed no significant associations with handgrip strength. Isotemporal substitution analyses indicated that reallocating short periods of time from sedentary behaviour to MVPA (e.g., 10–20 min/day) was associated with higher handgrip strength, whereas the inverse reallocation was associated with lower handgrip strength (Supplementary Figures S6–S13).

**Table 4:** Associations of handgrip strength and accelerometry-measured sedentary time, PA levels and sleep duration using a compositional analysis approach in preschool children

Behaviours	Compositional analysis approach		
	Mean difference in handgrip strength (kg)	95% CI	*P* value
ST (min/day)	−0.84	−1.31, −0.36	< 0.001
LPA (min/day)	−0.48	−1.23, 0.27	0.21
MVPA (min/day)	1.22	0.81, 1.62	< 0.001
Sleep duration (min/day)	0.1	−0.37, 0.57	0.689

Compositional analysis approach: adjusted for age, sex, BMI, mother education, father education and accelerometer wear time. β: unstandardized Beta. ST: Sedentary time; LPA: Light Physical Activity; MVPA: Moderate-to-Vigorous Physical Activity. Handgrip Strength was expressed in kilograms (kg) in all models.

**Table 5:** Associations of handgrip strength and accelerometry-measured sedentary behaviour, PA levels using a compositional analysis approach in preschool boys’ and girls’

Behaviours	Compositional analysis approach					
	Boys			Girls		
	β	95% CI	P value	β	95% CI	P value
ST (min/day)	-0.90	1.54, -0.26	0.006	-0.73	-1.46, 0	0.049
LPA (min/day)	-0.10	-1.14, 0.95	0.858	-0.81	-1.9, 0.27	0.141
MVPA (min/day)	0.96	0.37, 1.54	<0.001	1.46	0.89, 2.02	<0.001
Sleep duration (min/day)	0.04	-0.6, 0.68	0.912	0.09	-0.63, 0.81	0.812

Compositional analysis approach: adjusted for age, sex, BMI, mother education, father education and accelerometer wear time. β: unstandardized Beta. ST: Sedentary time; LPA: Light Physical Activity; MVPA: Moderate-to-Vigorous Physical Activity. Handgrip Strength was expressed in kilograms (kg) in all models.

Associations between time-use composition and handgrip strength with additional sex-stratified are presented in Table 5. Among boys, a larger relative proportion of MVPA was positively associated with handgrip strength (β = 0.96 kg; 95% CI: 0.37, 1.54), whereas a greater proportion of ST was inversely associated (β = − 0.90 kg; 95% CI: − 1.54, − 0.26). LPA and sleep duration were not significantly related to strength. In girls, the association with MVPA was even more pronounced, such that each unit increase in the relative log-ratio of MVPA corresponded to a 1.46 kg (95% CI: 0.89, 2.02) higher handgrip strength. ST showed a negative association (β = − 0.73 kg; 95% CI: − 1.46, 0.00), while LPA and sleep again exhibited no significant associations.

**Fig. 3:**
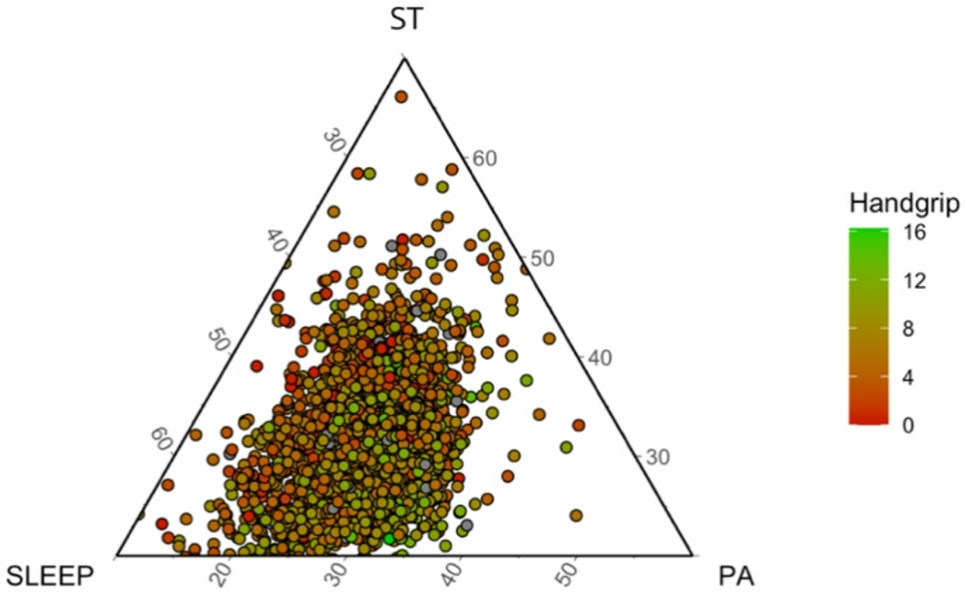
Ternary plot illustrating the daily time-use composition in sleep, ST, and PA, expressed as percentages of the 24-hour day. Each point represents one participant, and color indicates handgrip strength (in kilograms), with brighter green reflecting higher strength. The plot provides a visual representation of how participants’ time-use patterns relate to muscular strength levels. PA is the sum of LPA and MVPA. *ST* sedentary time, *PA* physical activity, *LPA* light physical activity, *MVPA* moderate-to-vigorous physical activity

Figure 3 shows the ternary plot representing the daily time-use composition in sleep duration, ST and PA levels, expressed as percentages of the 24-hour day. On average, preschool children spent 612.14 min/day in sleep duration (42.5%), 453.31 min/day in ST (31.5%), and 374.55 min/day in PA levels (26%), with PA being the sum of LPA and MVPA (318.08 and 56.47 min/day, respectively). Also, Fig. 3 shows the estimated relative change in handgrip strength when redistributing time between sleep duration, ST, LPA and MVPA.

## Discussion

The main aims of this study were to establish normative reference values for handgrip strength in a large sample of preschool children aged 3 to 5 years from the Stockholm region, and to examine the associations between handgrip strength and device-measured ST, PA levels and sleep duration. First, we propose the first handgrip strength normative reference values for Swedish preschool children. Median handgrip strength increased from 4.08 to 7.42 kg in boys and from 3.45 to 6.87 kg in girls between ages 3 and 5 years. Although the relative increase across ages was slightly greater among girls, boys were consistently stronger than girls at all ages. Another key finding was that handgrip strength was associated with the daily composition of time spent in movement behaviours; we found that MVPA was strongly and positively associated with handgrip strength, while ST showed an inverse association. No associations were observed for LPA or sleep duration.

The normative reference values for handgrip strength in Swedish preschool children developed in this study offer a valuable resource for understanding muscular strength development at an early age [6]. These data represent the most detailed reference values available internationally for this age group, with percentiles provided by sex, age, and hand dominance. Roriz De Oliveira et al. [22] provided reference values for Portuguese children aged 6 to 10, showing similar sex differences. However, the broader age range in that study and the lack of data for younger children highlight the importance of generating age-specific and population-specific normative reference values for the preschool age. Most previous studies have focused on children aged 4 to 5 years, with very limited data available for 3-year-olds; only one previous study from Spain has included this younger age group [9.] Using the PREFIT protocol in our study allows direct comparison and potential pooling with such data, which is important for establishing international reference values. Our findings suggest that Swedish preschool children have similar or slightly higher handgrip strength values compared to those reported in Spain, but further research is needed to explore these differences across countries. Therefore, future studies that establish preschool children’s handgrip strength should consider country differences, family social status, genetic and measure protocol. Recent methodological work has emphasized the importance of accounting for body size when interpreting handgrip strength across populations with differing growth patterns and socioeconomic contexts. Accordingly, we conducted exploratory allometric analyses and derived size-normalized reference values based on height and body mass following the framework proposed by Nevill et al. [28] As this framework was originally developed and validated in older children and adolescents, these findings should be interpreted with caution when applied to preschool-aged children. These supplementary analyses provide complementary information to the absolute normative values and may facilitate comparisons across populations with different anthropometric and socioeconomic profiles.

The inclusion of 3-year-old children in the present study provides novel and needed insights into muscular development from the earliest preschool years. Reference values for handgrip strength in the current study compared to reference standards from the Spanish study [9] suggest our values were consistently higher across ages 3 to 5 in both preschool boys and preschool girls. Reference values were broadly similar to those reported in Spanish preschool children, although differences varied by age. For example, the median in Swedish boys increased from 4.0 kg at age 3 to 7.4 kg at age 5, whereas Spanish values ranged from 3.9 kg to 8.4 kg across the same ages, suggesting that cross-country differences are not consistent across the age range [9]. A possible explanation for these differences between populations may lie in secular trends in muscle strength. This is supported by the findings of a recent international review that reported an overall improvement in handgrip strength in children over the past 50 years, with increases of approximately 24% in mean strength between 1967 and 2017 [11]. Although these trends have been documented primarily in older children and adolescents, they may be present also in younger populations, including preschool children.

An additional contribution of this study is the application of compositional data analysis (CoDA) to the 24-hour movement behaviour composition (ST, LPA, MVPA, and sleep duration) in relation to handgrip strength in preschool children. Among all the associations examined, MVPA showed a consistent positive association with handgrip strength. These findings are consistent with a recent systematic review, which concluded that handgrip strength in children appear to be a good indicator of MVPA [11]. A possible explanation for the negative association between ST and handgrip strength may be reduced exposure to active play and movement opportunities [23]. Similar findings were reported by Leppänen et al. (2016), [24] who noted that ST may displace PA, which is more strongly linked to muscular fitness in preschool children. In contrast, no significant associations were observed between handgrip strength and either LPA or sleep duration. A possible explanation is that LPA may not provide sufficient intensity, in contrast to MVPA, to stimulate improvements in handgrip strength among preschool children [24]. Similarly, no significant association was found between sleep duration and handgrip strength. Although sleep plays a fundamental role in overall child development and health, its direct impact on the development of muscle strength in early childhood may be limited, particularly when sleep duration is within the recommended levels for this age group. This lack of association is consistent with Abdeta et al. (2025) [25] who also reported no significant association between sleep duration and muscular strength in 3- and 4-year-old children.

### Practical Implications

The results of this study highlight the importance of nation-, age- and sex-specific percentiles for handgrip strength in preschool children. These reference values represent a valuable tool for identifying children with low muscular strength during a key developmental stage. Health and education professionals can use these percentiles to support early detection of low handgrip strength by providing targeted interventions. Using explicit, age- and sex-specific cut-points reduces subjectivity and ensures that mild–moderate weakness is not overlooked during routine health or school screenings. Furthermore, given the strong association observed between MVPA and handgrip strength, our findings highlight the importance of promoting physical activity from early childhood to support the development of muscular fitness [26]. Age-appropriate MVPA activities that involve upper-body engagement, such as climbing, pushing, or carrying, [27] should be promoted. Additionally, limiting prolonged ST remains essential to support optimal motor development and overall health. Collaboration among families, educators, and policymakers is key to creating supportive environments that foster PA and muscular strength in early childhood. From a practical perspective, percentile-based thresholds may help guide interpretation of handgrip strength in preschool children. Values below the 10th percentile may warrant closer attention and monitoring, while values below the 5th percentile may indicate a higher level of concern and justify further assessment, particularly when accompanied by other indicators of delayed motor development. Conversely, values at or above the 90th percentile may reflect advanced muscular strength for age and sex, potentially associated with higher habitual PA or favourable neuromuscular development. Importantly, these percentile-based thresholds are intended for screening and contextual interpretation only and should not be interpreted as diagnostic cut-points.

### Strengths and Limitations

This study has several strengths. To our knowledge, it is the first study in Northern Europe to provide sex, age, and hand-specific normative reference values for handgrip strength in a large sample of preschool children aged 3–5 years from the Stockholm region, including 3-year-olds, who are often underrepresented in research. Additionally, the use of compositional data analysis allowed for a more nuanced understanding of how different movement behaviours interact within the 24-hour day and their relationship with muscular strength. Finally, all outcomes were assessed using standardized and objective protocols, which increases the robustness and reproducibility of the results. The main limitation of this study is that, although associations were identified, the design does not allow for conclusions about causality. The relatively high proportion of children classified as underweight may partly reflect the use of international BMI reference standards, which are known to be conservative in early childhood and may classify a larger proportion of preschool children as underweight compared with other references. This should be considered when interpreting the distribution of weight-status categories in the present sample. Similarly, caution is required when interpreting normative cut-points; percentile-based thresholds are intended for screening purposes only and should not be interpreted as diagnostic cut-points. In addition, the normative reference values presented are based on absolute handgrip strength and are not adjusted for body size. Recent work has shown that allometric normalization using height and body mass may improve comparability across populations with different anthropometric and socioeconomic profiles, [28] and this should be considered when interpreting the present reference values. Another limitation is the lack of validated accelerometer cut-points for wrist-worn devices in preschool-aged children. Due to this, we applied cut-points developed in older children to classify physical activity intensities, which may not fully capture the movement patterns of this age group. Finally, the sample included a high proportion of parents with university education, which may limit the generalizability of the findings to populations with more diverse socioeconomic backgrounds.

## Conclusion

This study provides novel normative reference values for handgrip strength in Swedish preschool children aged 3 to 5 years and identified important associations between handgrip strength and the 24-hour movement behaviour composition, which identified that higher MVPA was strongly and positively associated with greater handgrip strength, whereas ST showed an inverse association. The percentiles developed in this study offer a practical tool for identifying preschool children with low muscular strength. Therefore, by enabling early identification of children with low muscular strength and offering context-specific reference percentiles, the reference values presented here can guide timely, targeted actions to support musculoskeletal development during a critical window of growth.

### Perspective

This study provides the first normative reference values for handgrip strength from Northern Europe, complementing previous data from Spain and enabling future pooled analyses [9]. By including 3-year-old children, it offers novel and needed insights into muscular development from the earliest preschool years. Our findings confirm that moderate-to-vigorous physical activity was strongly and positively associated with greater handgrip strength, whereas sedentary time showed an inverse association. These reference values offer a practical tool for identifying preschool children with low muscular strength and may guide timely, targeted actions to support musculoskeletal development during a critical window of growth.

## Supplementary Information

Supplementary Material 1.

## Data Availability

All data relevant to the study are included in the article or uploaded as supplementary information. Anonymized data should be available upon reasonable request.
