# Supplementary material for: Handgrip Strength Reference Values and Compositional Associations with Physical Activity in Early Childhood: A Large Sample Study of Swedish Preschoolers
Source: Sports Med Open. 2026 Feb 27;12:19. doi: 10.1186/s40798-026-00992-4 (PMC12949204; doi:10.1186/s40798-026-00992-4)
Supplement: Supplementary file 1 — Supplementary Material 1. [file 40798_2026_992_MOESM1_ESM.docx]

**Supplementary Material**

**Handgrip Strength Reference Values and Compositional Associations with Physical Activity in Early Childhood: A Large Sample Study of Swedish Preschoolers**

Ana Ramírez-Osuna^1^, Pablo Campos-Garzón^2,3^, Francisco Javier Huertas-Delgado^4^, Viktor H Ahlqvist^5,6^, Charlotte Wilén^2^, Pontus Henriksson^7^, Tommy R Lundberg^7,8^, Martin Neovius^2^, Micael Dahlen^10^, Daniel Berglind^2,9,10^

^1^Department of Physical Education and Sports, Faculty of Sport Sciences, Sport and Health University Research Institute (iMUDS), University of Granada, Granada, Spain.

^2^Department of Global Public Health, Karolinska Institutet, Stockholm, Sweden

^3^Faculty of Health Sciences, University of Lethbridge, Lethbridge, Alberta, Canada

^4^“La Inmaculada” Teacher Training Centre, University of Granada, 18013 Granada, Spain

^5^ Department of Biomedicine, Aarhus University, Aarhus, Denmark.

^6^ Institute of Environmental Medicine, Karolinska Insitutet, Stockholm, Sweden.

^7^ Department of Medical and Health Sciences, Linköping University, Linköping 581 83, Sweden.

^8^ Division of Clinical Physiology, Department of Laboratory Medicine, Karolinska Institute, Stockholm, Sweden

^9^ Unit of Clinical Physiology, Karolinska University Hospital, Stockholm, Sweden

^10^ Centre for Epidemiology and Community Medicine, Region Stockholm, Stockholm, Sweden

Corresponding to Pablo Campos-Garzón, Department of Global Public Health, Karolinska Institutet, Stockholm, Sweden. Faculty of Health Sciences, University of Lethbridge, Lethbridge, Alberta, Canada. (email: pablo.campos.garzon@ki.se).


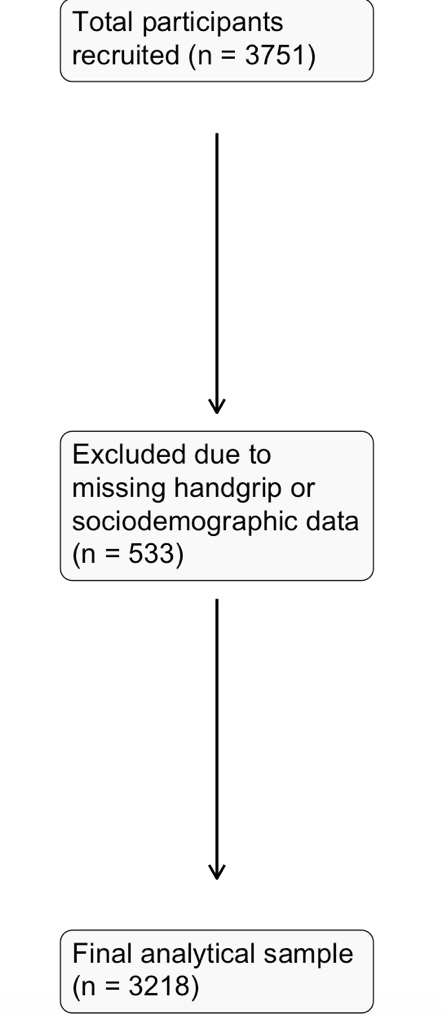


**Figure S1.** Flowchart of excluded participants

**Table S1.** Sensitivity analysis comparing included vs. excluded participants

| **Variable** | **Excluded** | **Included** | **P-value** |
| --- | --- | --- | --- |
|  | 533 | 3218 |  |
| **Age** | 4.5 (4.0, 5.0) | 4.5 (4.0, 5.0) | 0.2 |
| **Sex (n/%)** |  |  | 0.13 |
| Boy | 282 (53%) | 1656 (52%) |  |
| Girl | 251 (47%) | 1562 (48%) |  |
| **BMI (kg/m^2^)** | 16,1 (15.2, 17.1) | 16.1 (15.3, 17.1) | 0.5 |
| **MVPA (min/day)** | 55 (42, 71) | 58 (45, 73) | <0.001 |
| **Parental Education** | 297 (55%) | 1906 (59%) | <0.001 |
| **Maternal Education** | 236 (45%) | 1273 (42%) | <0.001 |
| Median (Q1, Q3); n (%)  Wilcoxon rank sum test; Pearson’s Chi-Squared Test | | | |

1. **Dominant Handgrip Strength Test**

**
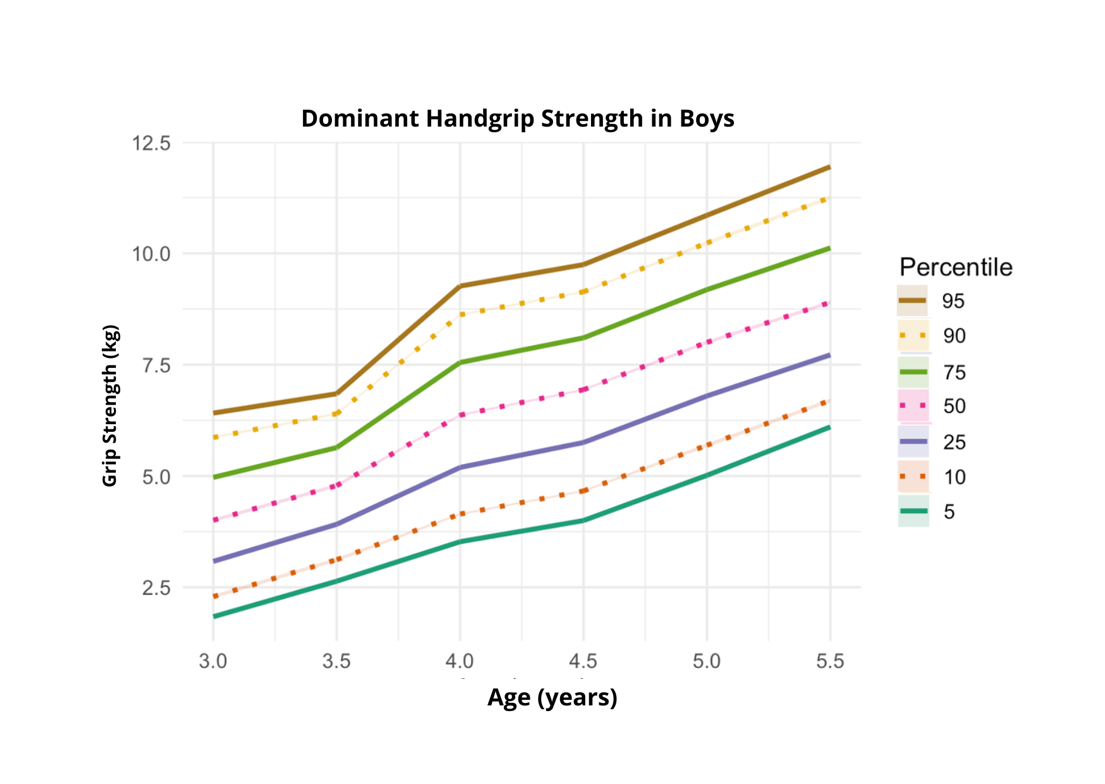
**

**Figure S2.** Percentile curves of handgrip strength by sex and age (a) dominant hand.

**
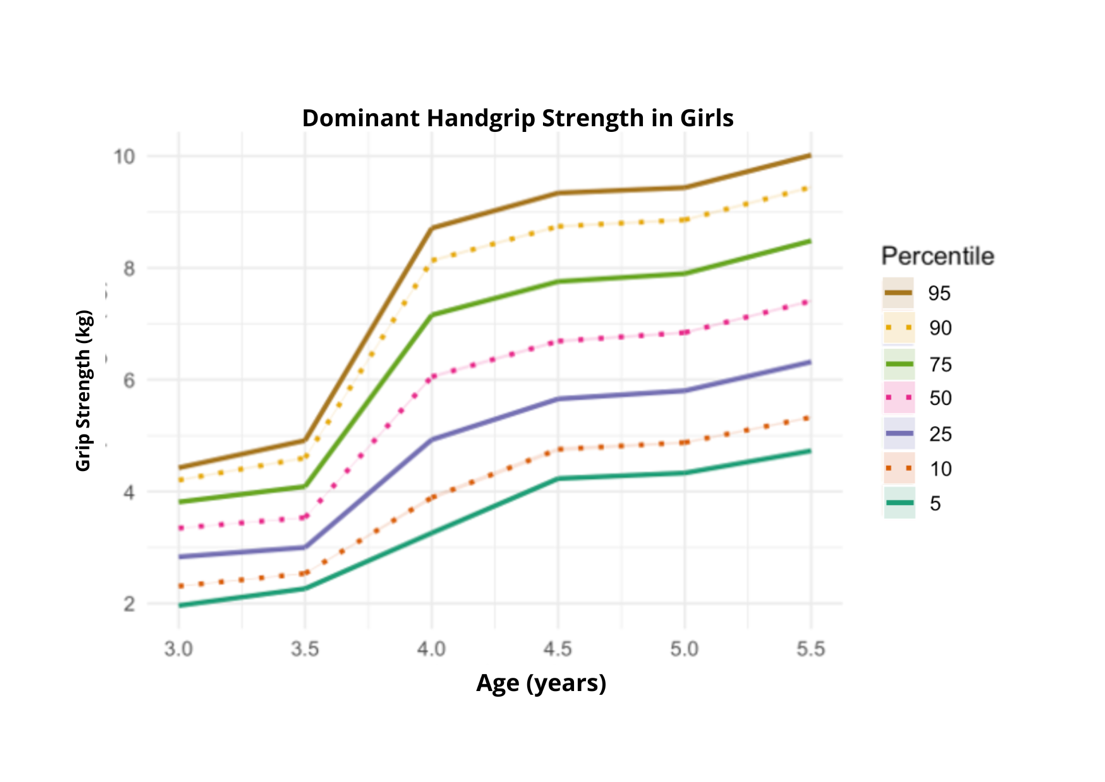
**

**Figure S3.** Percentile curves of handgrip strength by sex and age (a) dominant hand.

1. **No Dominant Handgrip Strength Test**

**
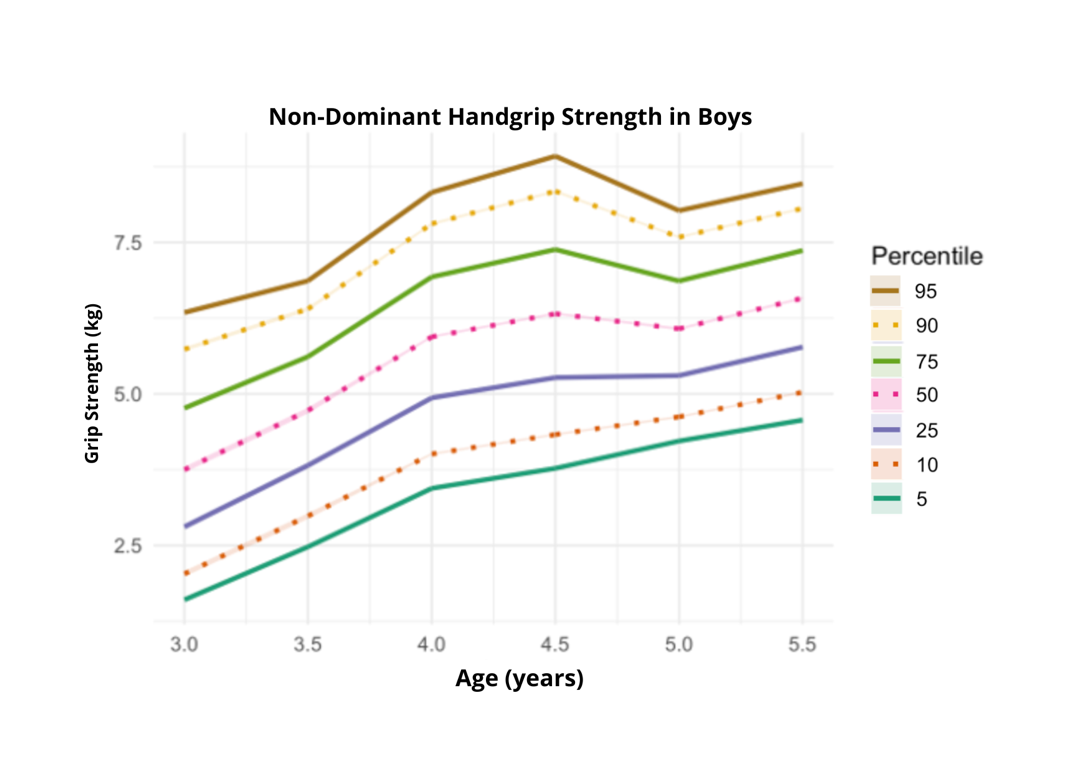
**

**Figure S4.** Percentile curves of handgrip strength by sex and age (b) no dominant hand.

**
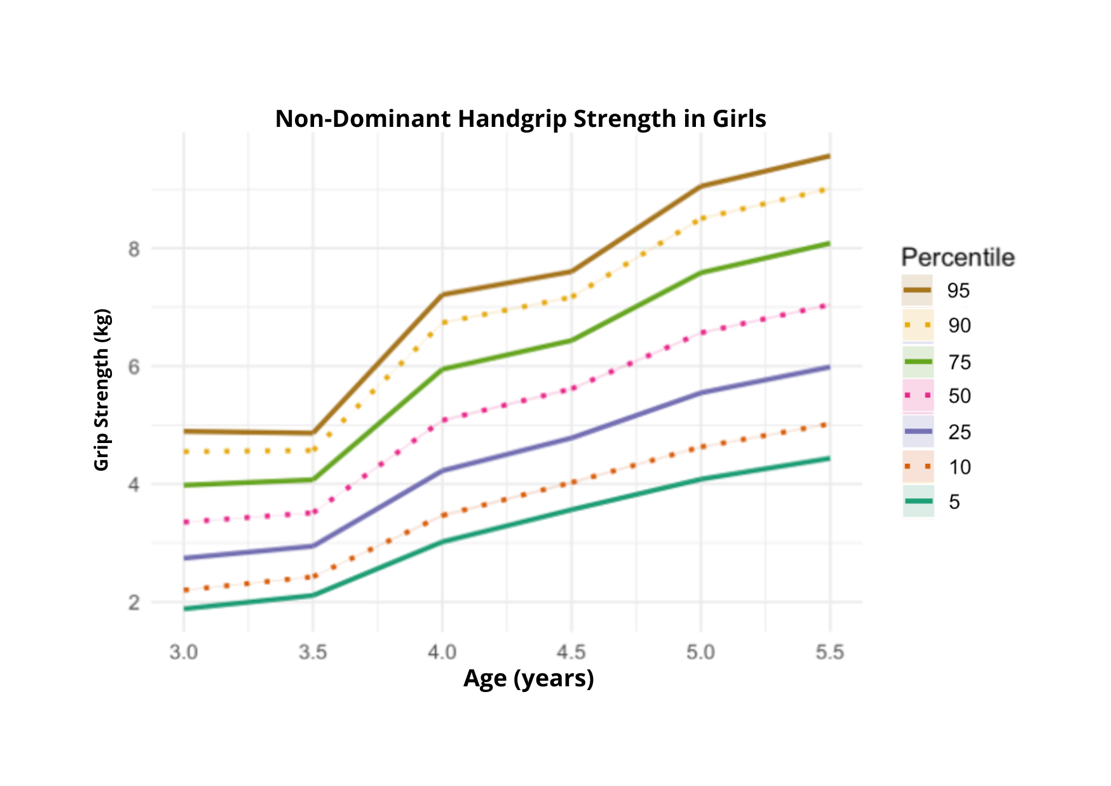
**

**Figure S5.** Percentile curves of handgrip strength by sex and age (b) no dominant hand.

| **Table S2.** Cut-off HGS measurements by side (dominant hand, non-dominant hand) and sex in all participants | | | | | | | |
| --- | --- | --- | --- | --- | --- | --- | --- |
| **PERCENTILES (GIRLS)** | | | | | | | |
|  | **5** | **10** | **25** | **50** | **75** | **90** | **95** |
| **D** | 2.88 (2.79,2.97) | 3.30 (3.19,3.42) | 4.50 (4.34,4.66) | 6.26 (6.10,6.41) | 7.61 (7.52,7.70) | 8.72 (8.46,8.97) | 9.30 (9.04,9.56) |
| **ND** | 2.70 (2.42,2.98) | 3.39 (3.20,3.58) | 4.11 (4.06,4.16) | 5.39 (5.21,5.56) | 6.86 (6.60,7.11) | 8.12 (7.99,8.26) | 8.49 (8.21,8.78) |
| **PERCENTILES (BOYS)** | | | | | | | |
|  | **5** | **10** | **25** | **50** | **75** | **90** | **95** |
| **D** | 3.58 (3.31,3.86) | 4.17 (3.88,4.46) | 5.30 (5.07,5.53) | 7.14 (6.28,7.46) | 8.59 (8.36,8.82) | 9.89 (9.63,10.16) | 10.89 (10.57,11.20) |
| **ND** | 3.35 (3.11,3.59) | 3.88 (3.61,4.16) | 4.93 (4.90,4.97) | 5.99 (5.89,6.08) | 6.91 (6.90,6.93) | 7.79 (7.69,7.89) | 8.21 (8.03,8.39) |
| Data are presented in kilograms (kg) for dominant hand and non-dominant hand. P10: 10^th^ percentiles; other percentiles are abbreviated accordingly. D=Dominant hand, ND= Non-dominant hand*.* Values in parentheses indicate the corresponding 95% confidence intervals (95% CI) | | | | | | | |

| **Table S3.**Reference standards of hand grip strength (kg) in boy’s preschool children | | | | | | | | |
| --- | --- | --- | --- | --- | --- | --- | --- | --- |
| **PERCENTILES (BOYS)** | | | | | | | | |
|  |  | **5 (95% CI)** | **10 (95% CI)** | **25 (95% CI)** | **50 (95% CI)** | **75 (95% CI)** | **90 (95% CI)** | **95 (95% CI)** |
| **3** | **D** | 1.84 (1.80,1.88) | 2.29 (2.25,2.33) | 3.08 (3.04,3.12) | 4.01 (3.97,4.04) | 4.97 (4.94,5.00) | 5.86 (5.84,5.89) | 6.41 (6.39,6.43) |
|  | **ND** | 1.60 (1.56,1.65) | 2.03 (1.99,2.08) | 2.81 (2.76,2.86) | 3.75 (3.70,3.80) | 4.77 (4.72,4.81) | 5.74 (5.70,5.77) | 6.34 (6.32,6.37) |
| **3.5** | **D** | 2.64 (2.60,2.68) | 3.12 (3.08,3.16) | 3.92 (3.88,3.96) | 4.78 (4.75,4.82) | 5.64 (5.61,5.67) | 6.40 (6.37,6.42) | 6.85 (6.83,6.87) |
|  | **ND** | 2.48 (2.44,2.52) | 2.99 (2.94,3.03) | 3.82 (3.77,3.87) | 4.73 (4.68,4.78) | 5.62 (5.57,5.66) | 6.40 (6.37,6.43) | 6.87 (6.84,6.89) |
| **4** | **D** | 3.52 (3.50,3.55) | 4.15 (4.12,4.17) | 5.19 (5.17,5.22) | 6.37 (6.34,6.39) | 7.55 (7.52,7.57) | 8.62 (8.60,8.64) | 9.26 (9.24,9.29) |
|  | **ND** | 3.44 (3.43,3.46) | 4.01 (3.99,4.03) | 4.94 (4.92,4.96) | 5.94 (5.93,5.96) | 6.93 (6.91,6.95) | 7.81 (7.78,7.83) | 8.32 (8.30,8.34) |
| **4.5** | **D** | 4.00 (3.98,4.02) | 4.66 (4.64,4.69) | 5.75 (5.73,5.78) | 6.94 (6.91,6.96) | 8.10 (8.08,8.13) | 9.14 (9.11,9.16) | 9.75 (9.73,9.77) |
|  | **ND** | 3.77 (3.76,3.79) | 4.33 (4.32,4.35) | 5.27 (5.25,5.29) | 6.32 (6.31,6.35) | 7.38 (7.36,7.41) | 8.34 (8.32,8.37) | 8.92 (8.90,8.94) |
| **5** | **D** | 5.02 (4.97,5.06) | 5.69 (5.65,5.73) | 6.80 (6.76,6.84) | 8.0(7.96,8.04) | 9.18 (9.15,9.22) | 10.2(10.2,10.3) | 10.9 (10.8,10.9) |
|  | **ND** | 4.22 (4.21,4.24) | 4.62 (4.61,4.64) | 5.30 (5.28,5.32) | 6.07 (6.05,6.10) | 6.86 (6.84,6.88) | 7.59 (7.57,7.61) | 8.02 (8.00,8.04) |
| **5.5** | **D** | 6.10 (6.06,6.15) | 6.70 (6.65,6.74) | 7.72(7.68,7.76) | 8.90 (8.86,8.94) | 10.1(10.1,10.2) | 11.3 (11.2,11.3) | 11.9 (11.9,12.0) |
|  | **ND** | 4.57 (4.56,4.59) | 5.03 (5.01,5.05) | 5.77 (5.75,5.79) | 6.58 (6.56,6.60) | 7.37 (7.35,7.39) | 8.06 (8.04,8.08) | 8.47 (8.45,8.49) |
| Data are presented for years of age, which correspond to dominant hand and non-dominant hand. P10: 10^th^ percentiles; other percentiles are abbreviated accordingly. D=Dominant hand, ND= Non-dominant hand*.* | | | | | | | | |

| **Table S4.** Reference standards of hand grip strength (kg) in girl’s preschool children | | | | | | | | |
| --- | --- | --- | --- | --- | --- | --- | --- | --- |
| **PERCENTILES (GIRLS)** | | | | | | | | |
|  |  | **5 (95% CI)** | **10 (95% CI)** | **25 (95% CI)** | **50 (95% CI)** | **75 (95% CI)** | **90 (95% CI)** | **95 (95% CI)** |
| **3** | **D** | 1.96 (1.95,1.98) | 2.31 (2.30,2.32) | 2.83 (2.82,2.84) | 3.35 (3.34,3.36) | 3.81 (3.80,3.83) | 4.20 (4.18,4.22) | 4.42 (4.40,4.45) |
|  | **ND** | 1.88 (1.87,1.89) | 2.20 (2.19,2.21) | 2.74 (2.73,2.75) | 3.35 (3.35,3.36) | 3.98 (3.97,3.98) | 4.55 (4.55,4.55) | 4.89 (4.89,4.90) |
| **3.5** | **D** | 2.26 (2.25,2.28) | 2.53 (2.52,2.55) | 3.00 (2.99,3.01) | 3.54 (3.53,3.55) | 4.09 (4.08,4.10) | 4.60 (4.58,4.62) | 4.91 (4.89,4.94) |
|  | **ND** | 2.11 (2.10,2.12) | 2.43 (2.41,2.44) | 2.94 (2.93,2.96) | 3.51 (3.50,3.52) | 4.07 (4.07,4.08) | 4.57 (4.57,4.57) | 4.86 (4.86,4.87) |
| **4** | **D** | 3.26 (3.21,3.30) | 3.89 (3.85,3.93) | 4.93 (4.89,4.96) | 6.05 (6.02,6.08) | 7.16 (7.13,7.18) | 8.13 (8.10,8.16) | 8.71 (8.68,8.74) |
|  | **ND** | 3.02 (2.99,3.04) | 3.47 (3.44,3.49) | 4.22 (4.20,4.25) | 5.08 (5.05,5.10) | 5.95 (5.92,5.97) | 6.73 (6.71,6.75) | 7.21 (7.19,7.23) |
| **4.5** | **D** | 4.23 (4.19,4.28) | 4.76 (4.72,4.79) | 5.66 (5.62,5.69) | 6.69 (6.66,6.72) | 7.76 (7.73,7.78) | 8.74 (8.71,8.77) | 9.34 (9.31,9.37) |
|  | **ND** | 3.57 (3.54,3.59) | 4.02 (4.00,4.05) | 4.78 (4.76,4.81) | 5.61 (5.59,5.64) | 6.43 (6.41,6.46) | 7.17 (7.15,7.19) | 7.60 (7.59,7.62) |
| **5** | **D** | 4.33 (4.31,4.35) | 4.88 (4.86,4.90) | 5.80 (5.78,5.82) | 6.84 (6.82,6.87) | 7.90 (7.87,7.92) | 8.86 (8.83,8.88) | 9.44 (9.41,9.46) |
|  | **ND** | 4.08 (4.06,4.10) | 4.63 (4.61,4.65) | 5.55 (5.53,5.56) | 6.56 (6.54,6.58) | 7.58 (7.56,7.60) | 8.50 (8.48,8.52) | 9.05 (9.03,9.07) |
| **5.5** | **D** | 4.73 (4.71,4.75) | 5.33 (5.31,5.35) | 6.32 (6.30,6.34) | 7.41 (7.38,7.43) | 8.48 (8.46,8.51) | 9.45 (9.42,9.47) | 10.00 (9.99,10.00) |
|  | **ND** | 4.44 (4.42,4.45) | 5.02 (5.00,5.04) | 5.99 (5.97,6.00) | 7.04 (7.02,7.06) | 8.08 (8.06,8.11) | 9.01 (8.99,9.04) | 9.57 (9.54,9.59) |
| Data are presented for years of age, which correspond to dominant hand and non-dominant hand. P10: 10^th^ percentiles; other percentiles are abbreviated accordingly. D=Dominant hand, ND= Non-dominant hand*.* | | | | | | | | |


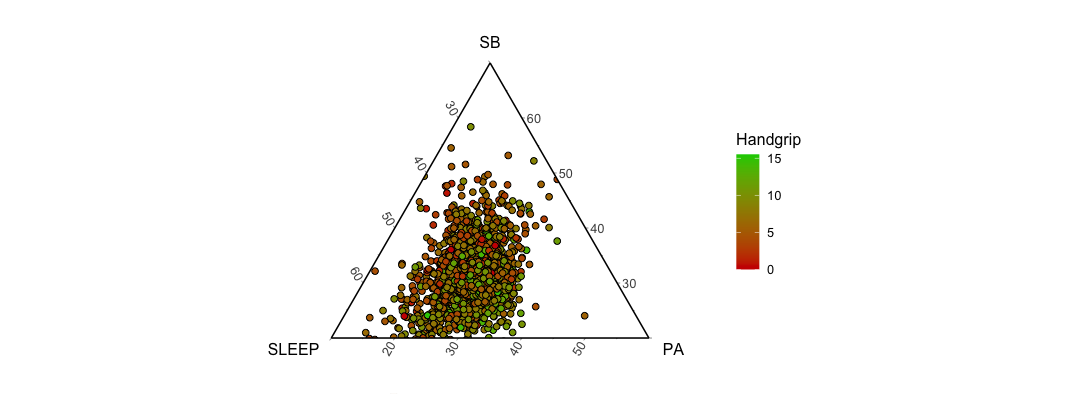


**Figure S6.** Ternary plot illustrating the daily time-use composition in sleep, ST,PA, expressed as percentages of the 24-hour day in preschool boys. Each point represents one participant, and colour indicates handgrip strength (in kilograms), with darker green reflecting higher strength. The plot provides a visual representation of how participants' time-use patterns relate to muscular strength levels. PA is the sum of light and moderate-to-vigorous physical activity.


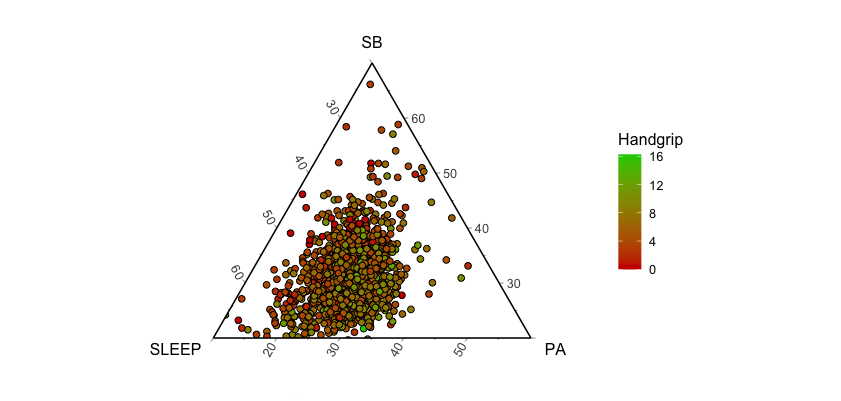


**Figure S7.** Ternary plot illustrating the daily time-use composition in sleep, ST), and PA, expressed as percentages of the 24-hour day in preschool girls. Each point represents one participant, and colour indicates handgrip strength (in kilograms), with darker green reflecting higher strength. The plot provides a visual representation of how participants' time-use patterns relate to muscular strength levels. PA is the sum of light and moderate-to-vigorous physical activity.


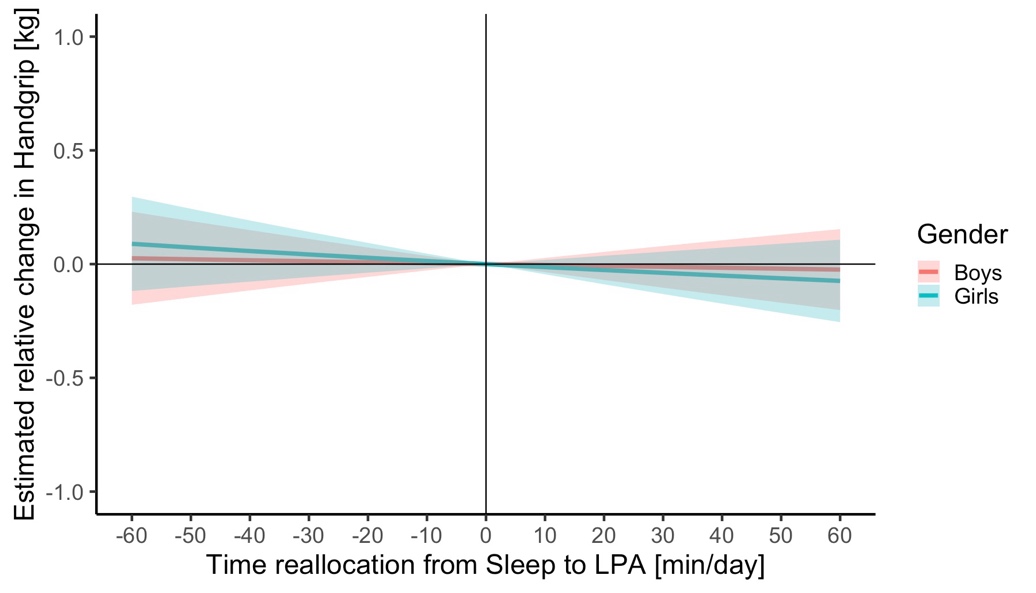


**Figure S8.** Pairwise cross-sectional associations from Sleep to Light Physical Activity in preschool boys and girls.


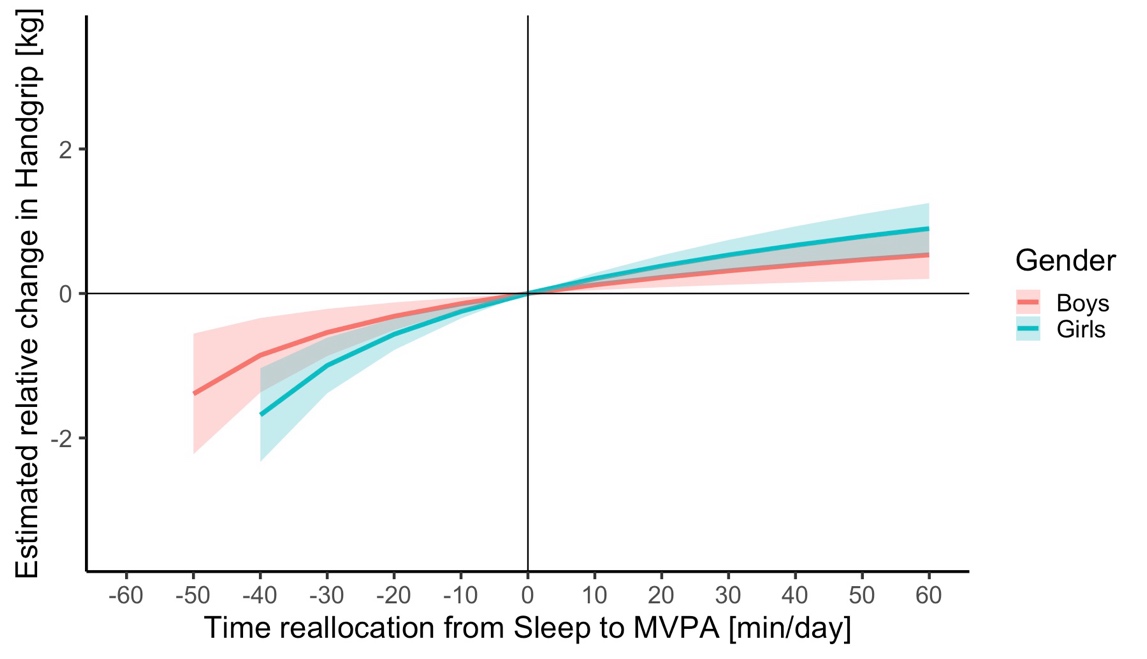


**Figure S9.** Pairwise cross-sectional associations from Sleep to Moderate-to-Vigorous Physical Activity in preschool boys and girls.


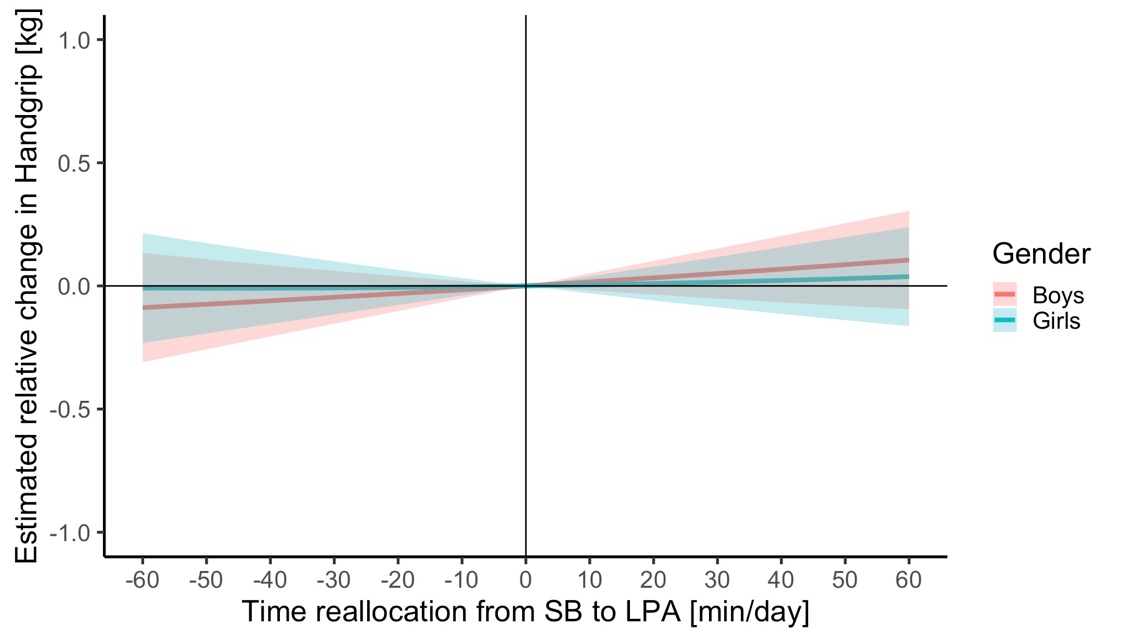


**Figure S10.** Pairwise cross-sectional associations from Sedentary Time to Light Physical Activity in preschool boys and girls.


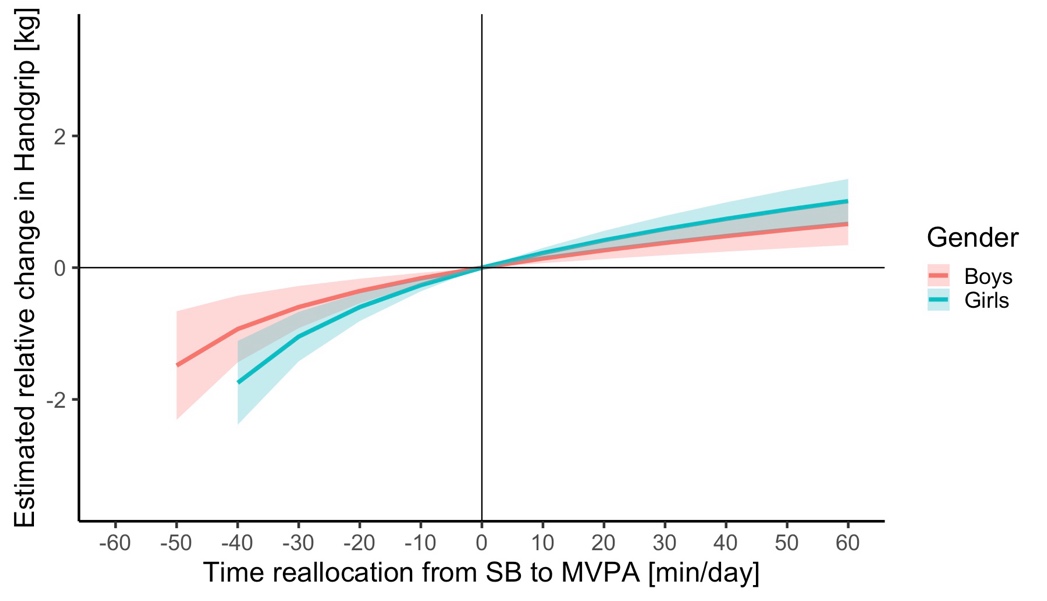


**Figure S11.** Pairwise cross-sectional associations from Sedentary Time to Moderate-to-Vigorous Physical Activity in preschool boys and girls.


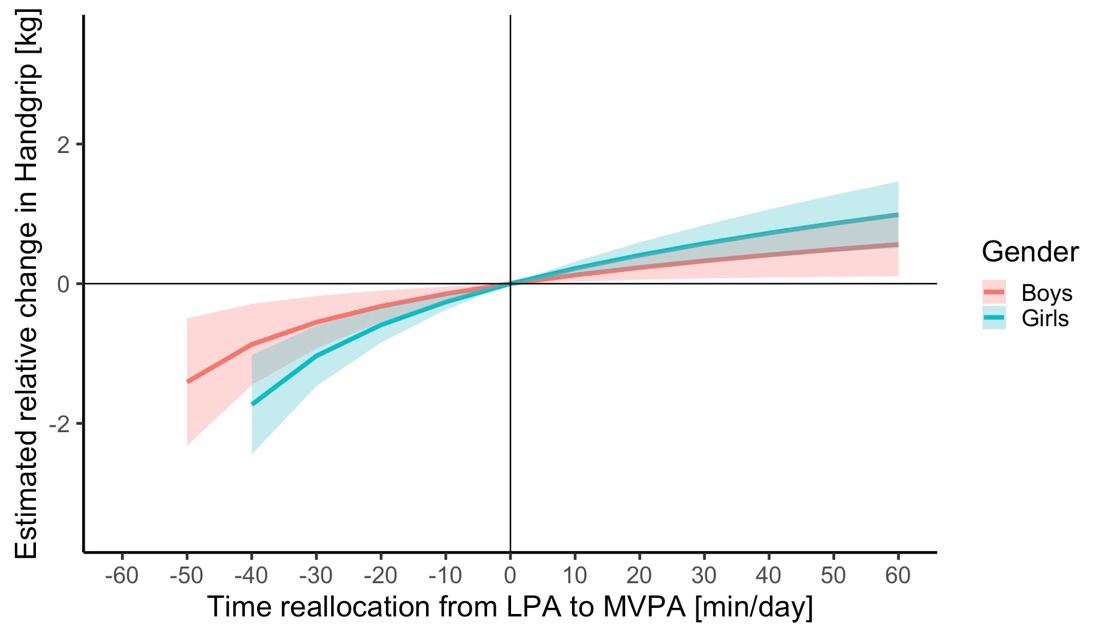


**Figure S12.** Pairwise cross-sectional associations from Light Physical Activity to Moderate-to-Vigorous Physical Activity in preschool boys and girls.


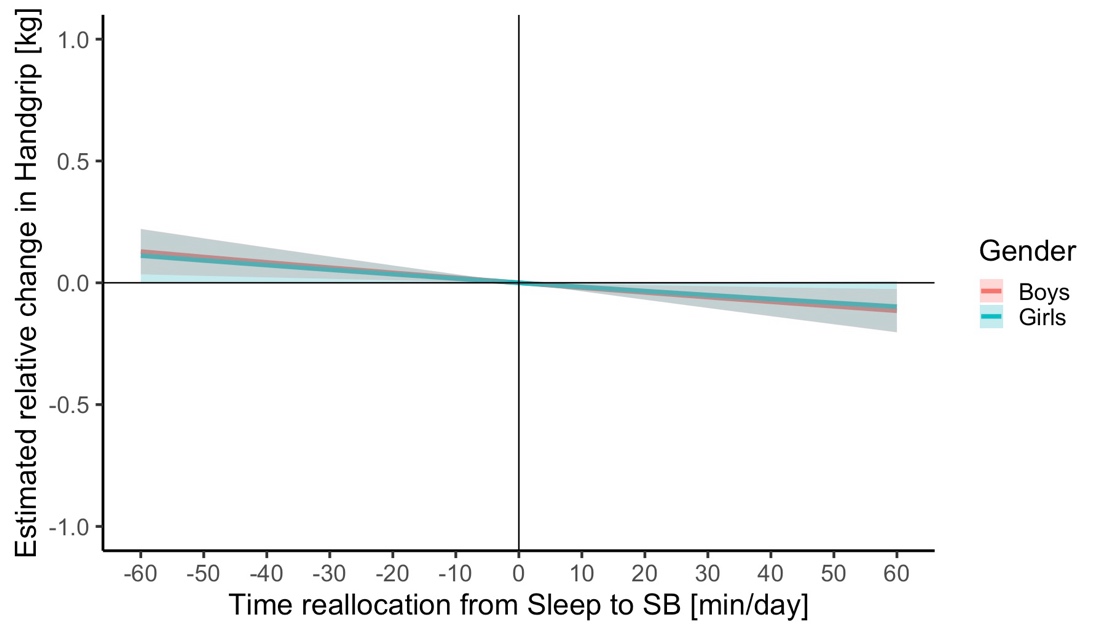


**Figure S13.** Pairwise cross-sectional associations from Sleep to Sedentary Time in preschool boys and girls.

| **Table S5. Exploratory allometric scaling of handgrip strength** | | | | | |
| --- | --- | --- | --- | --- | --- |
|  | **N** | **Height exponent (b)** | **Height exponent (SEE)** | **Mass exponent (b)** | **Mass exponent (SEE)** |
| Boys | 1656 | 2.49 | 0.33 | 0.52 | 0.11 |
| Girls | 1562 | 3.33 | 0.39 | 0.36 | 0.12 |
| All | 3218 | 2.95 | 0.25 | 0.44 | 0.08 |
| Scaling exponents were derived from log–log mixed-effects models with handgrip strength as the dependent variable and height and body mass as predictors, including a random intercept for school. Values are presented as regression coefficients (b) and their standard errors of the estimate (SEE). | | | | | |

| **Table S6.** Age- and sex-specific percentiles of size-normalized (kg/m^2^ * kg^0.333^) handgrip strength in preschool boys | | | | | | |
| --- | --- | --- | --- | --- | --- | --- |
|  | **P10** | **P25** | **P50** | **P75** | **P90** |  |
| **3.0** | 0.74 | 1.19 | 1.61 | 2.06 | 2.47 |  |
| **3.5** | 1.05 | 1.43 | 1.87 | 2.31 | 2.68 |  |
| **4.0** | 1.17 | 1.72 | 2.08 | 2.56 | 3.01 |  |
| **4.5** | 1.18 | 1.64 | 2.12 | 2.58 | 2.90 |  |
| **5.0** | 1.62 | 1.95 | 2.37 | 2.76 | 3.06 |  |
| **5.5** | 1.76 | 2.10 | 2.51 | 2.81 | 3.26 |  |
| Handgrip strength was normalized using the allometric “generalizable” ratio proposed by Nevill et al. (2025): **HGS_norm = HGS / (height² · mass^0.333),** where height is in meters and mass in kilograms. Values are presented as percentiles (P10–P90). | | | | | | |

| **Table S7.** Age- and sex-specific percentiles of size-normalized (kg/m^2^ * kg^0.333^) handgrip strength in preschool girls | | | | | |  |
| --- | --- | --- | --- | --- | --- | --- |
|  | **P10** | **P25** | **P50** | **P75** | **P90** | |
| **3.0** | 0.45 | 0.90 | 1.41 | 1.94 | 2.25 | |
| **3.5** | 0.74 | 1.19 | 1.66 | 2.06 | 2.48 | |
| **4.0** | 0.99 | 1.46 | 1.90 | 2.31 | 2.70 | |
| **4.5** | 1.25 | 1.65 | 2.01 | 2.40 | 2.73 | |
| **5.0** | 1.48 | 1.76 | 2.12 | 2.52 | 2.83 | |
| **5.5** | 1.53 | 1.86 | 2.25 | 2.61 | 2.96 | |
| Handgrip strength was normalized using the allometric “generalizable” ratio proposed by Nevill et al. (2025): **HGS_norm = HGS / (height² · mass^0.333),** where height is in meters and mass in kilograms. Values are presented as percentiles (P10–P90). | | | | | |  |
